# Effects of Coulomb Blockade on the Charge Transport through the Topological States of Finite Armchair Graphene Nanoribbons and Heterostructures

**DOI:** 10.3390/nano13111757

**Published:** 2023-05-29

**Authors:** David M. T. Kuo

**Affiliations:** 1 Department of Electrical Engineering, National Central University, Chungli 320, Taiwan, China; mtkuo@ee.ncu.edu.tw; 2 Department of Physics, National Central University, Chungli 320, Taiwan, China

**Keywords:** armchair graphene heterostructure, topological states, Coulomb blockade, thermoelectric properties, negative differential conductance, current rectification, Pauli spin blockade

## Abstract

In this study, we investigate the charge transport properties of semiconducting armchair graphene nanoribbons (AGNRs) and heterostructures through their topological states (TSs), with a specific focus on the Coulomb blockade region. Our approach employs a two-site Hubbard model that takes into account both intra- and inter-site Coulomb interactions. Using this model, we calculate the electron thermoelectric coefficients and tunneling currents of serially coupled TSs (SCTSs). In the linear response regime, we analyze the electrical conductance ($G_{e}$), Seebeck coefficient (*S*), and electron thermal conductance ($\kappa_{e}$) of finite AGNRs. Our results reveal that at low temperatures, the Seebeck coefficient is more sensitive to many-body spectra than electrical conductance. Furthermore, we observe that the optimized *S* at high temperatures is less sensitive to electron Coulomb interactions than $G_{e}$ and $\kappa_{e}$. In the nonlinear response regime, we observe a tunneling current with negative differential conductance through the SCTSs of finite AGNRs. This current is generated by electron inter-site Coulomb interactions rather than intra-site Coulomb interactions. Additionally, we observe current rectification behavior in asymmetrical junction systems of SCTSs of AGNRs. Notably, we also uncover the remarkable current rectification behavior of SCTSs of 9-7-9 AGNR heterostructure in the Pauli spin blockade configuration. Overall, our study provides valuable insights into the charge transport properties of TSs in finite AGNRs and heterostructures. We emphasize the importance of considering electron–electron interactions in understanding the behavior of these materials.

## 1. Introduction

The study of two-dimensional (2D) materials has gained significant attention since the discovery of graphene in 2004 [1,2,3,4]. Although graphene has limited applications in optical, semiconductor, and thermoelectric devices due to its gapless semi-metal nature, other 2D materials, such as $MoS_{2}$ and $WSe_{2}$, have shown potential for use in transistors and optoelectronics due to their direct band gaps. Low electron mobility and high contact resistance are two of the main challenges that need to be overcome to improve the performance of two-dimensional (2D) electronic devices, such as those made of $MoS_{2}$ and $WSe_{2}$ [5]. In recent years, researchers have explored the use of heterostructures made of 2D materials to improve the performance of quantum devices with ballistic transport [6].

To implement high-power output quantum devices, it is crucial to reduce the high contact resistance of 2D materials caused by contacted metallic electrodes [6]. On the other hand, certain low-power output quantum devices, such as single-electron transistors [7], single-photon emitters [8,9,10], spin-current conversion devices [11], single-quantum-dot heat engines [12], and solid-state quantum bits [13,14,15,16,17,18,19,20,21], require high contact resistances. Hence, 2D material nanostructures with small dielectric constants may have promising applications in low-power quantum devices. The exploration of electronic structures with deep energy levels that are well-separated from the band states in 2D material nanostructures is crucial for their development [22,23,24,25,26,27,28] since these quantum devices are operated on the basis of few discrete states that are well-separated from other continuous states to reduce charge transport density. A variety of graphene nanoribbons (GNRs) have been extensively studied, including armchair GNRs (AGNRs) [22], AGNR heterostructures [23], cove-edged zigzag GNRs [24], and chevron GNRs [25]. These GNRs have demonstrated the ability to exhibit diverse electronic topological states (TSs) based on their width, edge shape, and end terminations. The controllable manipulation of topological invariants in materials is a highly pursued research area. For instance, the utilization of electric fields and lattice strains has been explored to modulate TSs [26,27,28].

Significant progress has been made in the fabrication of graphene nanoribbons (GNRs) and heterostructures using the bottom-up synthesis technique [13,14,15,16,17,18,19,20,21]. The topological states (TSs) of the end zigzag edges of semiconducting AGNRs and interface states of AGNR heterostructures are well-separated from the conduction and valence subbands [19,20]. Therefore, these TSs may have potential for realizing low-power devices operating at room temperature. Due to graphene’s small dielectric constant, electron Coulomb interactions are expected to be significant in the TSs of finite AGNRs and heterostructures [29]. Investigating the effects of electron Coulomb interactions on low-power devices made of GNRs is desirable [30,31,32,33]. However, to date, there has been a lack of theoretical and experimental analysis concerning the Coulomb blockade effect in charge transport through serially coupled TSs (SCTSs) of AGNRs and AGNR heterostructures [34,35,36,37,38]. While various systems involving GNRs have exhibited topological phases [22,23,24,25,26,27,28], the utilization of AGNRs and AGNR heterostructures holds particular advantages for the realization of low-power quantum devices and circuits. This advantage arises from their symmetrical end-edge structures, which can be easily connected to line-contacted electrodes using current bottom-up synthesis techniques [38].

**Figure 1:**
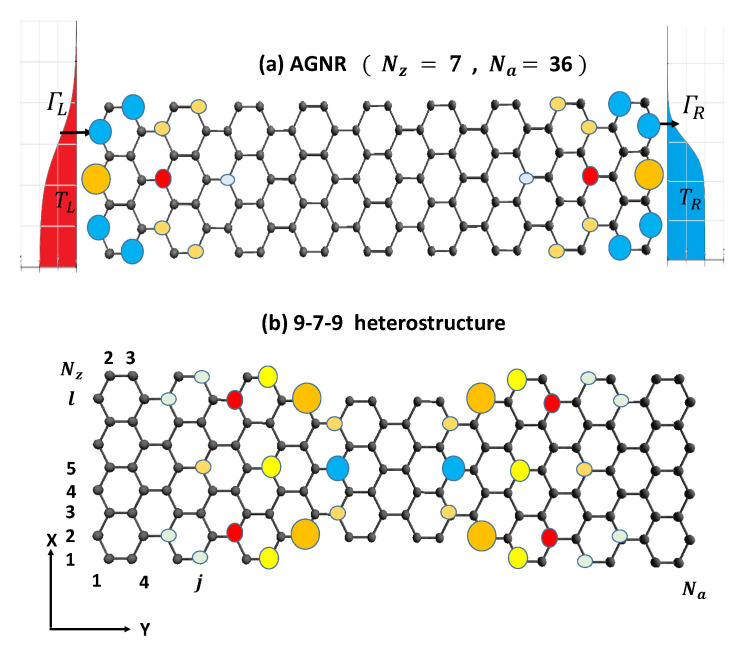
(**a**) Schematic diagram of an AGNR connected to electrodes. The tunneling rate of electrons between the left (right) electrode and the leftmost (rightmost) atoms of the AGNR is denoted by $\Gamma_{L}$ ($\Gamma_{R}$), respectively. The temperature of the left (*L*) and right (*R*) electrodes is represented by $T_{L}$ and $T_{R}$, respectively. The charge density of the localized zigzag edge state with energy ε=9.087 meV is shown for an AGNR with size characterized by $\left(N_{z},N_{a}\right)=\left(7,36\right)$. (**b**) Schematic lattice structure of AGNR heterostructures formed by three segments. Each segment is characterized by $\left(N_{z}=9\left(7\right),N_{a}=12\right)$. Note that $N_{a}=12$ corresponds to three unit cells (u.c.). The charge density of the topological state with energy ε=0.12156 eV is plotted for a 9/7/9 AGNR heterostructure. The radius of the circle represents the intensity of the charge density.

This study aims to investigate the charge transport mechanisms of SCTSs in two distinct systems: the end zigzag edge states of finite AGNRs and the topologically protected interface states of AGNR heterostructures coupled to leads, as shown in Figure 1a,b, respectively. A two-site model is employed to accurately represent the electrical conductance spectra resulting from charge transport through their SCTSs. A two-site Hubbard model with intra- and inter-site Coulomb interactions and Green’s function techniques are used to reveal the effects of Coulomb blockade on the charge transport of SCTS. In the linear response regime, we calculate the electrical conductance ($G_{e}$), Seebeck coefficient (*S*), and electron thermal conductance ($\kappa_{e}$) of AGNRs’ SCTSs. The Seebeck coefficient shows greater sensitivity to many-body spectra than electrical conductance at low temperatures. At high temperatures, electron Coulomb interactions do not affect the optimized values of *S*. In the nonlinear response regime, we observe a tunneling current exhibiting negative differential conductance (NDC). NDC arises due to inter-site electron Coulomb interactions in the absence of bias-dependent orbital offset. Moreover, in the asymmetrical tunneling junction systems of finite AGNRs, we observe current rectification behavior due to inter-site electron Coulomb interactions. Since the wave functions of TSs are far away from the contacted electrodes, 9-7-9 AGNR heterostructures can be readily set up in the Pauli spin blockade configuration. As a result, the tunneling current in a certain applied bias direction is strongly suppressed due to SCTSs that are highly occupied by two electron triplet states. This current rectification feature in the PSB configuration is very useful for spin-current conversion applications [11].

## 2. Calculation Methods

To model the transport properties of finite AGNRs and heterostructures connected to the electrodes shown in Figure 1, it is a good approximation to employ a tight-binding model with one $p_{z}$ orbital per atomic site to describe the electronic states [39,40,41]. The Hamiltonian of the nano-junction system depicted in Figure 1, including two different AGNR structures, can be written as $H=H_{0}+H_{AGNR}$ [42], where

(1) $$\begin{array}{ccc} H_{0} & = & \sum_{k}\epsilon_{k}a_{k}^{†}a_{k}+\sum_{k}\epsilon_{k}b_{k}^{†}b_{k}\\ & + & \sum_{\ell }\sum_{k}V_{k,\ell ,j}^{L}d_{\ell ,j}^{†}a_{k}+\sum_{\ell }\sum_{k}V_{k,\ell ,j}^{R}d_{\ell ,j}^{†}b_{k}+H.c. \end{array}$$

The first two terms of Equation (1) describe the free electrons in the left (*L*) and right (*R*) electrodes. $a_{k}^{†}$ ($b_{k}^{†}$) creates an electron with wave number *k* and energy $\epsilon_{k}$ in the left (right) electrode. $V_{k,\ell ,j=1}^{L}$ ($V_{k,\ell ,j=N_{z}\left(N_{a}\right)}^{R}$) describes the coupling between the left (right) lead with its adjacent atom in the *ℓ*-th row. The Hamiltonian for AGNRs can be expressed as:

(2) $$\begin{array}{c} H_{AGNR}=\sum_{\ell ,j}E_{\ell ,j}\;d_{\ell ,j}^{†}d_{\ell ,j}-\sum_{\ell ,j}\sum_{\ell^{\prime },j^{\prime }}t_{\left(\ell ,j\right),\left(\ell^{\prime },j^{\prime }\right)}d_{\ell ,j}^{†}d_{\ell^{\prime },j^{\prime }}-\sum_{\ell^{\prime },j^{\prime }}\sum_{\ell ,j}t_{\left(\ell^{\prime },j^{\prime }\right),\left(\ell ,j\right)}d_{\ell^{\prime },j^{\prime }}^{†}d_{\ell ,j}, \end{array}$$

where $E_{\ell ,j}$ is the on-site energy for the $p_{z}$ orbital in the *ℓ*-th row and *j*-th column. Here, the spin-orbit interaction is neglected. $d_{\ell ,j}^{†}\left(d_{\ell ,j}\right)$ creates (destroys) one electron at the atom site labeled by ( *ℓ*, *j* ) where *ℓ* and *j*, respectively, are the row and column indices as illustrated in Figure 1. $t_{\left(\ell ,j\right),\left(\ell^{\prime },j^{\prime }\right)}$ describes the electron hopping energy from site ( $\ell^{\prime }$, $j^{\prime }$ ) to site ( *ℓ*, *j* ). The tight-binding parameters used for AGNRs is $E_{\ell ,j}=0$ for the on-site energy and $t_{\left(\ell ,j\right),\left(\ell^{\prime },j^{\prime }\right)}=t_{pp\pi }=2.7$ eV for the nearest neighbor hopping strength.

To study the transport properties of an AGNR junction connected to electrodes, it is convenient to use the Keldysh Green function technique [42]. In the linear response regime, the electrical conductance ($G_{e}$), Seebeck coefficient (*S*), and electron thermal conductance ($\kappa_{e}$) are given by $G_{e}=e^{2}L_{0}$, $S=-L_{1}/\left(eTL_{0}\right)$, and $\kappa_{e}=\frac{1}{T}\left(L_{2}-L_{1}^{2}/L_{0}\right)$, respectively. Here, $L_{n}$ (n=0,1,2) is defined as

(3) $$L_{n}=\frac{2}{h}\int d\varepsilon \;T_{LR}\left(\varepsilon \right){\left(\varepsilon -\mu \right)}^{n}\frac{\partial f(\varepsilon )}{\partial \mu }.$$

The Fermi distribution function of electrodes at equilibrium temperature *T* and chemical potential μ is given by $f\left(\varepsilon \right)=1/\left(exp^{\left(\varepsilon -\mu \right)/k_{B}T}+1\right)$. The transmission coefficient $T_{LR}\left(\varepsilon \right)$, as shown in Equation (3), is a critical factor in electron transport between the left (*L*) and right (*R*) electrodes. The numerical code can be used to calculate $T_{LR}\left(\varepsilon \right)$, which is given by $T_{LR}\left(\varepsilon \right)=4Tr\left[\Gamma_{L}\left(\varepsilon \right)G^{r}\left(\varepsilon \right)\Gamma_{R}\left(\varepsilon \right)G^{a}\left(\varepsilon \right)\right]$. Here, $\Gamma_{L}\left(\varepsilon \right)$ and $\Gamma_{R}\left(\varepsilon \right)$ represent the tunneling rate (in energy units) at the left and right leads, respectively. Additionally, $G^{r}\left(\varepsilon \right)$ and $G^{a}\left(\varepsilon \right)$ correspond to the retarded and advanced Green functions of the AGNR, respectively [43,44,45].

## 3. Results and Discussion

### 3.1. Charge Transport through a Finite Armchair Graphene Nanoribbon (AGNR)

**Figure 2:**
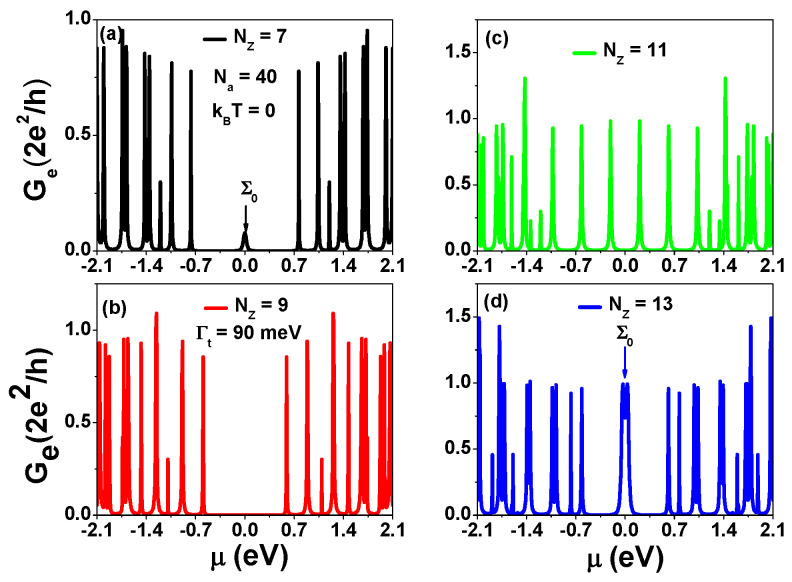
Electrical conductance ($G_{e}$) as a function of chemical potential (μ) for AGNRs with a fixed armchair direction width of $N_{a}=40$ ($L_{a}=4.12$ nm) and various widths ( $N_{z}$ ) at a tunneling rate of $\Gamma_{L}=\Gamma_{R}=\Gamma_{t}=90$ meV and zero temperature (T=0 K). (**a**) $N_{z}=7$, (**b**) $N_{z}=9$, (**c**) $N_{z}=11$, and (**d**) $N_{z}=13$.

The electronic behavior of AGNRs is determined by their widths, which follow the rule $N_{z}=3m+2$, $N_{z}=3m+1$, and $N_{z}=3m$, where *m* is an integer. AGNRs exhibit semiconducting behavior for $N_{z}=3m+1$ and $N_{z}=3m$, resulting in semiconducting phases for widths such as $N_{z}=7$, $N_{z}=9$, and $N_{z}=13$ with corresponding band gaps of 1.26 eV, 0.948 eV, and 0.714 eV, respectively, in the absence of electron Coulomb interactions [40,41]. Notably, AGNRs with $N_{z}=3m+2$ maintain their semiconducting phases, as determined by first-principle calculations in references [22,28]. However, the one-band tight-binding model still captures the main physics for the cases of $N_{z}=3m$ and $N_{z}=3m+1$, which is the primary focus of this article. To investigate charge transport through AGNRs with line contacted electrodes, as depicted in Figure 1a, we present the calculated electron conductance ($G_{e}$) as a function of μ at zero temperature for various $N_{z}$ values of finite AGNRs with $N_{a}=40$ in Figure 2. The magnitude of $G_{e}$ is given in units of the quantum conductance of $G_{0}=\frac{2e^{2}}{h}=1/\left(12.9\;K\Omega \right)=77.5$ μS. For the case of $N_{z}=7$ in Figure 2a, we observe a peculiar peak labeled by $\Sigma_{0}$ at the charge neutrality point (CNP) (μ=0). In contrast, the spectra around the CNP are vanishingly small for $N_{z}=9$, as seen in Figure 2b. The uniform peak separation in Figure 2c corresponds to the linear dispersion of AGNRs with a metallic phase at $N_{z}=11$. In particular, $\Sigma_{0}$ is split into two peaks around the CNP for $N_{z}=13$ in Figure 2d. The electrical conductance near the CNP is attributed to electron transport through the end zigzag edges of finite AGNRs. The wave functions of the left and right zigzag edges of AGNRs decay along the armchair directions, and their overlaps are vanishingly small in infinitely long AGNRs. When $N_{a}$ is finite, the zero mode energy levels are lifted due to the coupling between these two localized wave functions which can be regarded as a SCTS. The magnitude of the lifted energy level can be determined by the electron hopping strength $t_{LR}$ between the left and right zigzag edges [20]. In Figure 2, we have $\left|t_{LR}\right|=5.323$ meV, 0.1 meV, and 39.67 meV for $N_{z}=7$, $N_{z}=9$, and $N_{z}=13$, respectively. Since $\left|t_{LR}\right|/\Gamma_{t}\ll 1$, the peak of $\Sigma_{0}$ is degraded in the case of $N_{z}=9$. The results of Figure 2 indicate that $t_{LR}$ is significantly affected by the AGNR width, which is either $N_{z}=3m+1$ or $N_{z}=3m$. In Appendix A, we discuss the electronic structures of 9-7 AGNR superlattices and charge transport through the SCTSs of 9-7-9 AGNR heterostructures, which were proposed as useful quantum bits [20].

**Figure 3:**
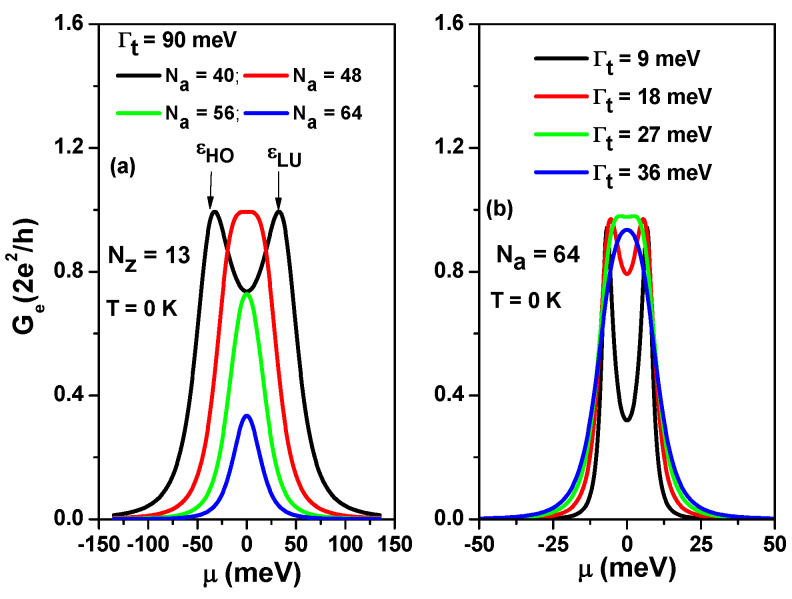
(**a**) Electrical conductance $G_{e}$ as a function of chemical potential μ for AGNRs of different lengths, while keeping the tunneling rate fixed at $\Gamma_{t}=90$ meV and the temperature at T=0 K. (**b**) Electrical conductance $G_{e}$ as a function of chemical potential μ for AGNRs with $N_{a}=64$ ($L_{a}=6.674$ nm), while varying the tunneling rate $\Gamma_{t}$ at T=0 K.

To better understand the transport behavior of the SCTSs, Figure 3a shows the calculated electrical conductance ($G_{e}$) as a function of μ for various $N_{a}$ at $\Gamma_{t}=90$ meV, T=0 K and $N_{z}=13$. As $N_{a}$ increases for a fixed $N_{z}=13$, $t_{LR}$ decreases, with values of 39.67 meV, 22.29 meV, 12.59 meV, and 7.13 meV for $N_{a}=40$, $N_{a}=48$, $N_{a}=56$, and $N_{a}=64$, respectively. As seen in Figure 3a, $\varepsilon_{HO}$ and $\varepsilon_{LU}$, which are the highest occupied molecular orbital (HOMO) and the lowest unoccupied molecular orbital (LUMO), respectively, can be resolved only for $N_{a}=40$. For $N_{a}=64$, the magnitude of electrical conductance is smaller than 0.4 $G_{0}$. To clarify the effect of contacted electrodes on the transport of the SCTS, we plot the calculated $G_{e}$ for various $\Gamma_{t}$ values for $N_{a}=64$ and $N_{z}=13$ in Figure 3b. The electrical conductance reaches the value of one quantum conductance $G_{0}$ at $\Gamma_{t}=27$ meV. When $\Gamma_{t}$ is smaller than 27 meV, the peaks $\varepsilon_{HO}$ and $\varepsilon_{LU}$ can be distinguished. The results shown in Figure 3 indicate that resolving the peaks of $\varepsilon_{HO}$ and $\varepsilon_{LU}$ is challenging, as it requires proper modulation of the $t_{LR}/\Gamma_{t}$ ratio.

**Figure 4:**
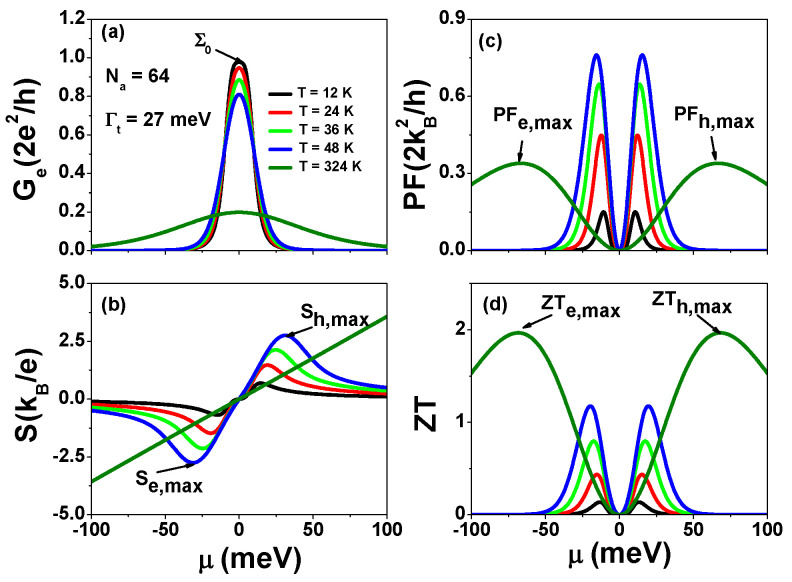
(**a**) Electrical conductance $G_{e}$, (**b**) Seebeck coefficient *S*, (**c**) power factor $PF=S^{2}G_{e}$, and (**d**) figure of merit $ZT=S^{2}G_{e}T/\left(\kappa_{e}+\kappa_{ph}\right)$ as functions of μ for AGNRs with $N_{z}=13$, $N_{a}=64$, and $\Gamma_{t}=27$ meV at various temperatures.

Up to now, it remains unclear about the thermoelectric properties of SCTSs [19,20,21]. In Figure 4, we present the calculated electrical conductance $G_{e}$, Seebeck coefficient *S*, power factor $PF=S^{2}G_{e}$, and thermoelectric figure of merit ZT as functions of chemical potential μ for various temperatures for AGNRs with $N_{z}=13$ and $N_{a}=64$. The spectra of $G_{e}$ exhibit a symmetrical function of μ, while *S* displays a bipolar behavior with respect to μ. At T=48 K, the maximum $S_{h(e),max}=\pm 2.753$. Notably, *S* can be well described by S=μ/T at room temperature (T=324 K), indicating that the thermoelectric properties of zigzag edge states of AGNRs with $N_{z}=13$ and $N_{a}=64$ resemble those of a single localized state at room temperature [46,47,48]. In Figure 4c, the maximum power factors ($PF_{h(e),max}$) are 0.76 and 0.339 for T=48 K and T=324 K, respectively. At T=324 K, $PF_{h(e),max}$ occurs at μ=±66.68 meV, with the ratio of $\mu /k_{B}T=\pm 2.46$. In ref. [46], the authors predicted that the optimized PF of a single energy level without energy level broadening satisfies $\mu /k_{B}T=\pm 2.4$. In Figure 4d, we calculated ZT using the equation $ZT=S^{2}G_{e}T/\left(\kappa_{e}+\kappa_{ph}\right)$. Here, we used a phonon thermal conductance value of $\kappa_{ph}=F_{s}*\frac{\pi^{2}k_{B}^{2}T}{3h}$, where we assumed a scattering factor of $F_{s}=0.1$. For the remainder of this article, we will use the same value of $\kappa_{ph}=F_{s}*\frac{\pi^{2}k_{B}^{2}T}{3h}$ with $F_{s}=0.1$. In a previous study [48], the authors proposed introducing defects in GNRs to increase phonon scattering while maintaining the electron transmission coefficient of topological states. More recently, we demonstrated [43] that zigzag edge states in AGNRs with vacancies remained robust. The maximum $ZT_{h(e),max}=1.966$ at T=324 K occurs at μ=±68.3 meV. Here and henceforth, *S* is expressed in units of $k_{B}/e=86.25\;\mu$V/K, and PF is in units of $2k_{B}^{2}/h=0.575$ pW/K${}^{2}$. The results of Figure 4 provide us with thermoelectric coefficients of the SCTS in the absence of electron Coulomb interactions.

### 3.2. Effective Hamiltonian and Formula for Tunneling Current

It is challenging to calculate the tunneling current through SCTSs in the Coulomb blockade region using either first-principle methods [22] or tight-binding models [44]. Hence, we introduce a two-site Hubbard model to clarify the thermoelectric coefficients and tunneling current of SCTSs in the Coulomb blockade region. The Hamiltonian of the two-site Hubbard model is given by

(4) $$\begin{array}{ccc} H_{2-site} & = & \sum_{j=L,R,\sigma }E_{j}c_{j,\sigma }^{†}c_{j,\sigma }-t_{LR}\;\left(c_{R,\sigma }^{†}c_{L,\sigma }+c_{L,\sigma }^{†}c_{R,\sigma }\right)\\ & + & \sum_{j=L,R}U_{j}\;n_{j,\sigma }n_{j,-\sigma }+\frac{1}{2}\sum_{j\neq \ell ,\sigma ,\sigma^{\prime }}U_{j,\ell }\;n_{j,\sigma }n_{\ell ,\sigma^{\prime }}, \end{array}$$

where $E_{j}$ represents the spin-independent energy level of the TSs, $U_{j}=U_{L(R)}=U_{0}$ and $U_{j,\ell }=U_{LR}=U_{1}$ denote the intra-site and inter-site Coulomb interactions, respectively, and $n_{j,\sigma }=c_{j,\sigma }^{†}c_{j,\sigma }$. $U_{0}$ and $U_{1}$ are calculated using $\frac{1}{4\pi \epsilon_{0}}\sum_{i,j}|\Psi_{L(R)}\left(r_{i}\right){|}^{2}{\left|\Psi_{L(R)}\left(r_{j}\right)\right|}^{2}\frac{1}{|r_{i}-r_{j}|}$ with the dielectric constant $\epsilon_{0}=4$, and $U_{cc}=4$ eV at i=j. $U_{cc}$ arises from the two-electron occupation in each $p_{z}$ orbital. $\Psi_{L(R)}\left(r_{i}\right)$ is the wave functions of TSs [29].

Based on the effective Hamiltonian given by Equation (4), we derive the tunneling current through the SCTSs coupled to the electrodes after tedious algebra [49]. We obtain the expression of tunneling current leaving from the left (right) electrode as

(5) $$\begin{array}{ccc} J_{L(R)}\left(V_{bias},T\right) & = & \frac{2e}{h}\int d\varepsilon \;T_{LR(RL)}^{2-site}\left(\varepsilon \right)\left[f_{L}\left(\varepsilon \right)-f_{R}\left(\varepsilon \right)\right]. \end{array}$$

The Fermi distribution function for the α-th electrode is denoted by $f_{\alpha }\left(\varepsilon \right)=1/\left(\exp\left[\left(\varepsilon -\mu_{\alpha }\right)/k_{B}T\right]+1\right)$, where $\mu_{L(R)}$ is the chemical potential of the left (right) electrode with an applied bias of $V_{bias}/2$ ($-V_{bias}/2$), such that $\mu_{L(R)}=\mu \pm eV_{bias}/2$. The transmission coefficient for charge transport through the SCTSs, denoted by $T_{LR}^{2-site}\left(\varepsilon \right)$, has a closed-form expression given by

(6) $$\begin{array}{ccc} & & T_{LR}^{2-site}\left(\epsilon \right)/\left(4t_{LR}^{2}\Gamma_{e,L}\Gamma_{e,R}\right)=\frac{P_{1}}{|\epsilon_{L}\epsilon_{R}-t_{LR}^{2}{|}^{2}}\\ & + & \frac{P_{2}}{|\left(\epsilon_{L}-U_{LR}\right)\left(\epsilon_{R}-U_{R}\right)-t_{LR}^{2}{|}^{2}}\\ & + & \frac{P_{3}}{|\left(\epsilon_{L}-U_{LR}\right)\left(\epsilon_{R}-U_{LR}\right)-t_{LR}^{2}{|}^{2}}\\ & + & \frac{P_{4}}{|\left(\epsilon_{L}-2U_{LR}\right)\left(\epsilon_{R}-U_{LR}-U_{R}\right)-t_{LR}^{2}{|}^{2}}\\ & + & \frac{P_{5}}{|\left(\epsilon_{L}-U_{L}\right)\left(\epsilon_{R}-U_{LR}\right)-t_{LR}^{2}{|}^{2}}\\ & + & \frac{P_{6}}{|\left(\epsilon_{L}-U_{L}-U_{LR}\right)\left(\epsilon_{R}-U_{R}-U_{LR}\right)-t_{LR}^{2}{|}^{2}}\\ & + & \frac{P_{7}}{|\left(\epsilon_{L}-U_{L}-U_{LR}\right)\left(\epsilon_{R}-2U_{LR}\right)-t_{LR}^{2}{|}^{2}}\\ & + & \frac{P_{8}}{|\left(\epsilon_{L}-U_{L}-2U_{LR}\right)\left(\epsilon_{R}-U_{R}-2U_{LR}\right)-t_{LR}^{2}{|}^{2}}, \end{array}$$

where $\epsilon_{L}=\varepsilon -E_{L}+i\Gamma_{e,L}$ and $\epsilon_{R}=\varepsilon -E_{R}+i\Gamma_{e,R}$. $\Gamma_{e,L(R)}$ is an effective tunneling rate for the left (right) TS coupled to the left (right) electrode. The transmission coefficient given by Equation (6) consists of eight terms, each corresponding to one of the eight possible configurations of the SCTS that an electron with spin σ from the left electrode may encounter. The probabilities of these configurations are as follows:

$$\begin{array}{ccc} P_{1} & = & 1-N_{L,\sigma }-N_{R,\sigma }-N_{R,-\sigma }+\langle n_{R,\sigma }n_{L,\sigma }\rangle \\ & + & \langle n_{R,-\sigma }n_{L,\sigma }\rangle +\langle n_{R,-\sigma }n_{R,\sigma }\rangle -\langle n_{R,-\sigma }n_{R,\sigma }n_{L,\sigma }\rangle \\ P_{2} & = & N_{R,\sigma }-\langle n_{R,\sigma }n_{L,\sigma }\rangle -\langle n_{R,-\sigma }n_{R,\sigma }\rangle \\ & + & \langle n_{R,-\sigma }n_{R,\sigma }n_{L,\sigma }\rangle \\ P_{3} & = & N_{R,-\sigma }-\langle n_{R,-\sigma }n_{L,\sigma }\rangle -\langle n_{R,-\sigma }n_{R,\sigma }\rangle \\ & + & \langle n_{R,-\sigma }n_{R,\sigma }n_{L,\sigma }\rangle \\ P_{4} & = & \langle n_{R,-\sigma }n_{R,\sigma }\rangle -\langle n_{R,-\sigma }n_{R,\sigma }n_{L,\sigma }\rangle \\ P_{5} & = & N_{L,\sigma }-\langle n_{R,\sigma }n_{L,\sigma }\rangle -\langle n_{R,-\sigma }n_{L,\sigma }\rangle \\ & + & \langle n_{R,-\sigma }n_{R,\sigma }n_{L,\sigma }\rangle \\ P_{6} & = & \langle n_{R,\sigma }n_{L,\sigma }\rangle -\langle n_{R,-\sigma }n_{R,\sigma }n_{L,\sigma }\rangle \\ P_{7} & = & \langle n_{R,-\sigma }n_{L,\sigma }\rangle -\langle n_{R,-\sigma }n_{R,\sigma }n_{L,\sigma }\rangle \\ p_{8} & = & \langle n_{R,-\sigma }n_{R,\sigma }n_{L,\sigma }\rangle , \end{array}$$

where the intra-site and inter-site two-particle correlation functions are denoted by $\langle n_{\ell ,-\sigma }n_{\ell ,\sigma }\rangle$ and $\langle n_{\ell ,\sigma }n_{j,\sigma }\rangle$ ($\langle n_{\ell ,-\sigma }n_{j,\sigma }\rangle$), respectively. $\langle n_{\ell ,-\sigma }n_{\ell ,\sigma }n_{j,\sigma }\rangle$ is the three-particle correlation function. These correlation functions can be solved self-consistently, and it should be noted that probability conservation is satisfied by $\sum_{m}P_{m}=1$.

The expression for $T_{LR}^{2-site}\left(\varepsilon \right)$ goes beyond not only mean field theory [38], but also our previous work [49], where we only considered one-particle occupation numbers and intra-site two-particle correlation functions. Theoretical analysis has shown that inter-site two-particle correlation functions play a significant role when inter-site Coulomb interactions are large ($U_{1}>t_{LR}$) [50]. To obtain $T_{RL}^{2-site}\left(\varepsilon \right)$, we can simply exchange the indices in Equation (6). Based on Equation (6), the expression for the electrical conductance at zero temperature for a 2-site model without Coulomb interactions is given by:

(7) $$\begin{array}{ccc} G_{e}\left(\mu \right) & = & G_{0}\frac{4\Gamma_{e,L}t_{LR}^{2}\Gamma_{e,R}}{|\left(\mu -E_{L}+i\Gamma_{e,L}\right)\left(\mu -E_{R}+i\Gamma_{e,R}\right)-t_{LR}^{2}{|}^{2}}. \end{array}$$

**Figure 5:**
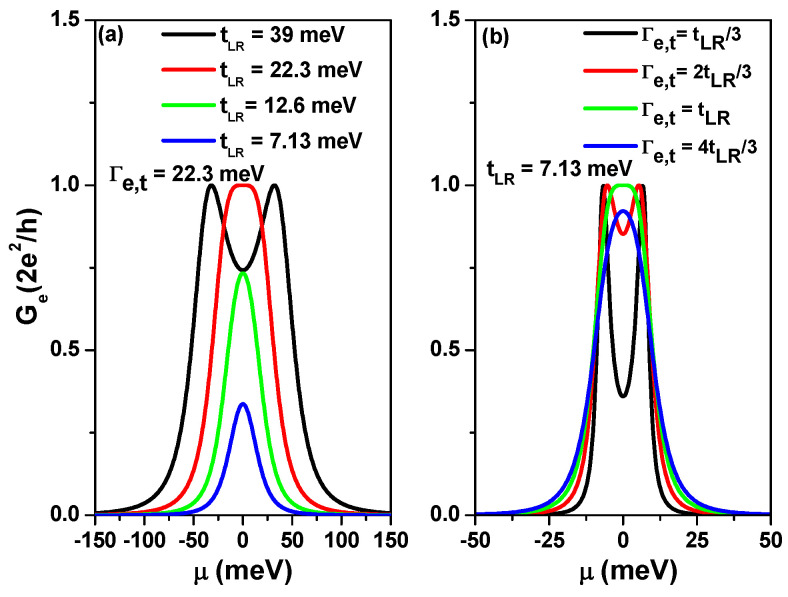
(**a**) Electrical conductance as a function of μ for various $t_{LR}$ values at zero temperature and $\Gamma_{e,t}=22.3$ meV and (**b**) electrical conductance as a function of μ for various $\Gamma_{e,t}$ values at zero temperature and $t_{LR}=7.13$ meV. The calculated lines are based on Equation (7).

Using Equation (7), we calculated $G_{e}$ for different values of $t_{LR}$ at $\Gamma_{e,L}=\Gamma_{e,R}=\Gamma_{e,t}=22.3$ meV in Figure 5a. The considered $t_{LR}$ values correspond to $N_{a}=40$, $N_{a}=48$, $N_{a}=56$, and $N_{a}=64$. Similarly, we calculated $G_{e}$ for various $\Gamma_{e,t}$ values at $t_{LR}=7.13$ meV in Figure 5b. It is worth noting that the curves shown in Figure 5a,b are identical to the curves of Figure 3a,b, respectively. These results in Figure 5 illustrate that charge transport through the SCTS can be well explained with a two-site model that introduces an effective tunneling rate $\Gamma_{e,t}$. In the following discussion, we will consider charge transport through the SCTSs in the presence of electron Coulomb interactions, both in the linear and nonlinear regimes.

### 3.3. Effects of Coulomb Blockade on Charge Transport through the Zigzag Edges of AGNRs

#### 3.3.1. Linear Response Regime

**Figure 6:**
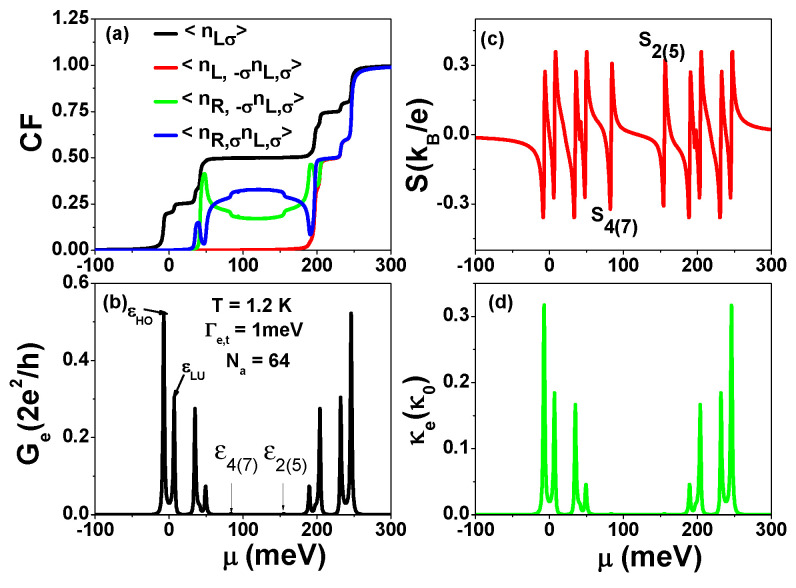
(**a**) Correlation functions (CF), (**b**) electrical conductance ($G_{e}$), (**c**) Seebeck coefficient *S* and (**d**) electron thermal conductance ($\kappa_{e}$) as functions of chemical potential μ at $\Gamma_{e,t}=1$ meV, and T=1.2 K. We have set $E_{j}=0$, $U_{0}=155$ meV, $U_{1}=42$ meV and $t_{LR}=7.13$ meV. These physical parameters correspond to AGNRs with $N_{a}=64$ and $N_{z}=13$.

**Figure 7:**
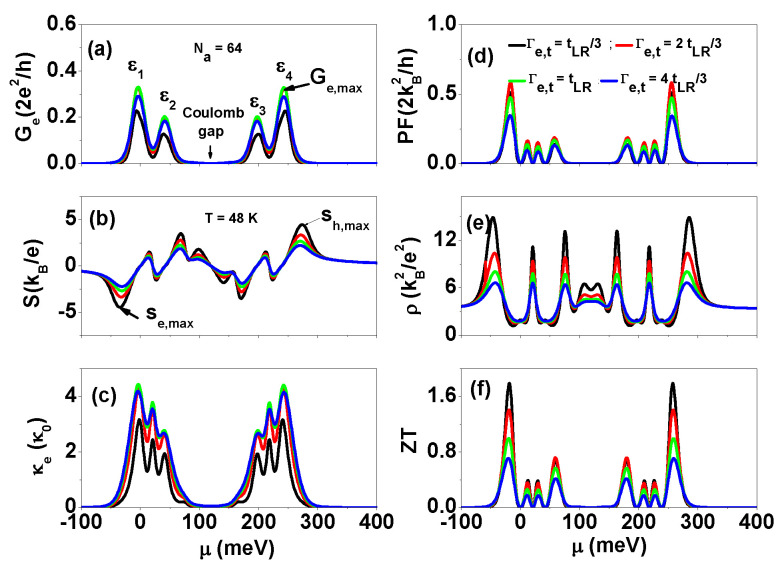
(**a**) Electrical conductance $G_{e}$, (**b**) Seebeck coefficient *S*, (**c**) electron thermal conductance $\kappa_{e}$, (**d**) power factor PF, (**e**) Lorenz number $\rho =\kappa_{e}/\left(TG_{e}\right)$ and (**f**) figure of merit ZT as functions of μ for various $\Gamma_{e,t}$ values at T=48 K. Other physical parameters are the same as those of Figure 6.

**Figure 8:**
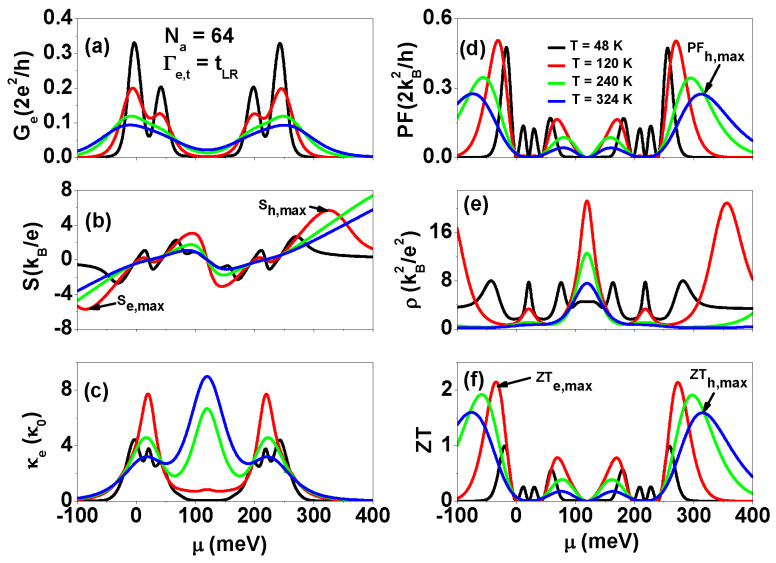
(**a**) Electrical conductance, (**b**) Seebeck coefficient, (**c**) electron thermal conductance, (**d**) power factor, (**e**) Lorenz number ρ, and (**f**) figure of merit ZT as functions of μ for different temperature values of AGNRs at $\Gamma_{e,t}=t_{LR}$. The physical parameters considered are the same as those in Figure 6.

In this subsection, we examine the effects of the Coulomb blockade on the charge transport through the end zigzag edges of AGNRs in the linear and nonlinear response regimes. We utilize Equations (3) and (6) to calculate the correlation functions (CF), electrical conductance $G_{e}$, Seebeck coefficient *S*, and electron thermal conductance $\kappa_{e}$ of the zigzag edge states as functions of μ at T=1.2 K and $\Gamma_{e,t}=1$ meV, and present the results in Figure 6. Figure 6a shows the electron single particle occupation number $N_{j,\sigma }=\langle n_{j,\sigma }\rangle$, which displays four major plateaus corresponding to the values of 1/4, 1/2, 3/4, and 1 (or total electron number $N_{T}=\sum_{j,\sigma }N_{j,\sigma }$ corresponding to 1, 2, 3, and 4). These plateaus arise due to electron Coulomb interactions. In contrast to the two-electron singlet state in each site $\langle n_{L,-\sigma }n_{L,\sigma }\rangle$ and $\langle n_{R,-\sigma }n_{R,\sigma }\rangle$, which occur at a large μ value near 197 meV, the inter-site triplet state $\langle n_{R,\sigma }n_{L,\sigma }\rangle$ and the inter-site singlet state $\langle n_{R,-\sigma }n_{L,\sigma }\rangle$ appear near μ≈42 meV. Figure 6b presents the spectra of $G_{e}$, which can reveal electron hopping strength ($t_{LR}=7.13$ meV), inter-site Coulomb interactions ($U_{1}=42$ meV), and intra-site Coulomb interactions ($U_{0}=155$ meV) due to low temperature and weak $\Gamma_{e,t}$. The asymmetrical electrical conductances labeled by $\varepsilon_{HO}$ and $\varepsilon_{LU}$ indicate that the probability of $P_{1}$ depends on the μ of the electrodes. In the Coulomb gap region ($N_{j,\sigma }=0.5$), there are two tiny peaks labeled by $\varepsilon_{4(7)}$ and $\varepsilon_{2(5)}$ corresponding to $P_{4(7)}$ and $P_{2}\left(P_{5}\right)$ channels. Because they are off-resonant channels as $E_{L}=E_{R}$, their electrical conductances are quite small. In the Pauli spin blockade configuration, they are in resonant channels, which will be discussed later. Figure 6c shows the spectra of Seebeck coefficient *S*, which can more clearly reveal the two tiny spectra of $G_{e}$ ($\varepsilon_{4(7)}$ and $\varepsilon_{2(5)}$) because $S=-\frac{\pi^{2}k_{B}^{2}T}{3e}\frac{1}{G_{e}\left(\mu \right)}\frac{\partial G_{e}\left(\mu \right)}{\partial \mu }$ at low temperatures. Figure 6d displays the spectra structure of electron thermal conductance $\kappa_{e}$, which is quite similar to $G_{e}$ spectra at low temperatures, whereas they are quite different at high temperatures (see results of Figure 7 and Figure 8). Note that $\kappa_{e}$ is in units of $\kappa_{0}=0.62\;nW/K$.

To clarify the contact properties in the presence of Coulomb interactions, we then calculated several thermoelectric quantities, including $G_{e}$, *S*, $\kappa_{e}$, PF, $\rho =\kappa_{e}/\left(TG_{e}\right)$, and ZT, as functions of μ for various $\Gamma_{e,t}$ at a temperature of 48K, and plotted the results in Figure 7. Consistent with the results in Figure 5, the maximum $G_{e}$ still occurred at the condition of $\Gamma_{e,t}=t_{LR}$, which is not affected by the intra-site and inter-site Coulomb interactions. At T=48 K, the $\varepsilon_{HO}$ and $\varepsilon_{LU}$ peaks are washed out in the spectra of $G_{e}$, whereas the Coulomb gap between $\varepsilon_{2}$ and $\varepsilon_{3}$ arising from intra-site Coulomb interactions remains. Although the spectra of *S* become more complex in the presence of electron Coulomb interactions, it does not significantly affect the maximum $S_{h(e),max}$. Comparing with the results of $S_{h(e),max}$ at T=48 K shown in Figure 4, the maximum $S_{h(e),max}$ has a value of ±2.684 at $\Gamma_{e,t}=t_{LR}$, which is almost the same as $S_{h(e),max}$ of Figure 4. The spectra of $\kappa_{e}$ show two main structures, each with three peaks, and an extra peak appears in the charge blockade region (as seen in the $G_{e}$ spectra) as the temperature increases. The maximum PF does not occur at $\Gamma_{e,t}=t_{LR}$, as seen in Figure 7d. The Lorenz number of charge transport through the zigzag edges does not satisfy the Wiedemann–Franz law $L_{z}=\kappa_{e}/\left(TG_{e}\right)=\frac{\pi^{2}k_{B}^{2}}{3e^{2}}$ in Figure 7e, indicating that $\kappa_{e}$ and $G_{e}$ may not be relevant thermoelectric quantities in discrete energy level systems. Figure 7f shows that the maximum ZT values prefer smaller $\Gamma_{e,t}$ and $\rho =\kappa_{e}/\left(TG_{e}\right)<L_{z}$.

To effectively apply heat engines [12], it is crucial to clarify the thermoelectric quantities over a wide temperature range. In Figure 8, we depict the behavior of $G_{e}$, *S*, $\kappa_{e}$, PF, ρ, and ZT as functions of μ for various temperatures at $\Gamma_{e,t}=t_{LR}$. It can be observed that the magnitude of $G_{e}$ reduces with increasing temperature. When the temperature is at 200 K and 324 K, the inter-site Coulomb interactions responsible for the structure in $G_{e}$ spectra are washed out. In Figure 8b, the sophisticated spectra of *S* become an N-shaped curve at room temperature. In Figure 8c, the highest $\kappa_{e}$ occurs at the middle Coulomb gap when the temperature is at 240 K and 324 K. This behavior can be understood using the expression for electron thermal conductance $\kappa_{e}=\frac{1}{T}\left(L_{2}-L_{1}^{2}/L_{0}\right)=\frac{L_{2}}{T}-S^{2}G_{e}T$. When μ is located at the middle Coulomb gap, the electron-hole balance requires *S* to be close to zero. Therefore, $\kappa_{e}$ is dominated by $\frac{L_{2}}{T}$, which generally has a significant contribution at high temperatures based on the thermionic procedure where μ does not align with resonant channels. This explains why $\kappa_{e}$ reaches its maximum value in the middle Coulomb gap. The maximum power factors are PF=0.476 and PF=0.274 for T=48 K and T=324 K, respectively. These values are lower than the maximum PF values in Figure 4c, attributed to the reduction in $G_{e}$ resulting from the Coulomb blockade. As seen in Figure 8e, $\rho =\kappa_{e}/\left(TG_{e}\right)$ displays temperature-dependent behavior. Clearly, the reduction in $G_{e}$ also suppresses the maximum ZT values. Nevertheless, the maximum $ZT_{h(e),\max}=1.6$ at T=324 K in the Coulomb blockade region still reaches 80% of $ZT_{h(e),\max}=1.966$ shown in Figure 4, without considering electron Coulomb interactions.

#### 3.3.2. Nonlinear Response Regime

**Figure 9:**
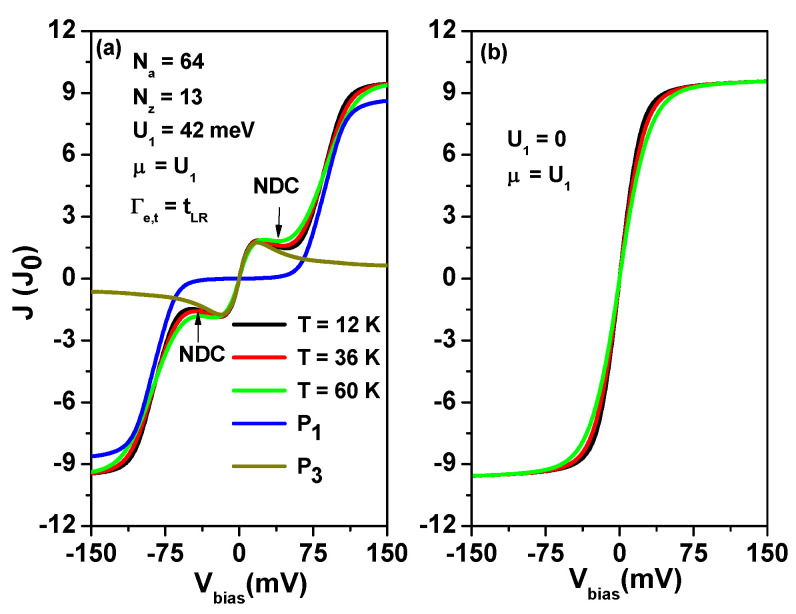
Tunneling current (*J*) of AGNRs with $N_{z}=13$ and $N_{a}=64$ as a function of applied bias for various temperatures at $\Gamma_{e,t}=t_{LR}$, with $t_{LR}=7.13$ meV and $U_{0}=155$ meV. (**a**) shows the case where $U_{1}=42$ meV, while (**b**) shows the case where $U_{1}=0$. We also set $E_{L}=E_{R}=0$, and $\mu =U_{1}$. Additionally, (**a**) includes the tunneling current calculated by the $P_{1}$ and $P_{3}$ channels at T=12 K. The tunneling current is reported in units of $J_{0}=0.773$ nA.

Recent experimental studies have reported negative differential conductance (NDC) in charge transport through the AGNR heterostructure tunneling junction [37,38]. However, the underlying mechanism for NDC at low bias remains unclear [37,38]. In this study, we propose a novel mechanism for NDC resulting from the inter-zigzag edge Coulomb interactions $U_{1}$ (or inter-site Coulomb interactions). By utilizing Equations (5) and (6), we calculate the tunneling current as a function of applied bias $V_{bias}$ for different temperatures, as shown in Figure 9a,b. We considered $U_{1}$ values of 42 meV and 0 meV for Figure 9a,b, respectively. In the forward (reversed) bias, the tunneling current is determined by the transmission coefficient of $T_{LR}^{2-site}\left(\varepsilon \right)$ ($T_{RL}^{2-site}\left(\varepsilon \right)$). As seen in Figure 9a, at low temperatures (T=12 K), the tunneling current in the low bias region decreases with increasing $V_{bias}$. This intriguing behavior demonstrates the NDC phenomenon, which is absent at higher temperatures.

**Figure A4:**
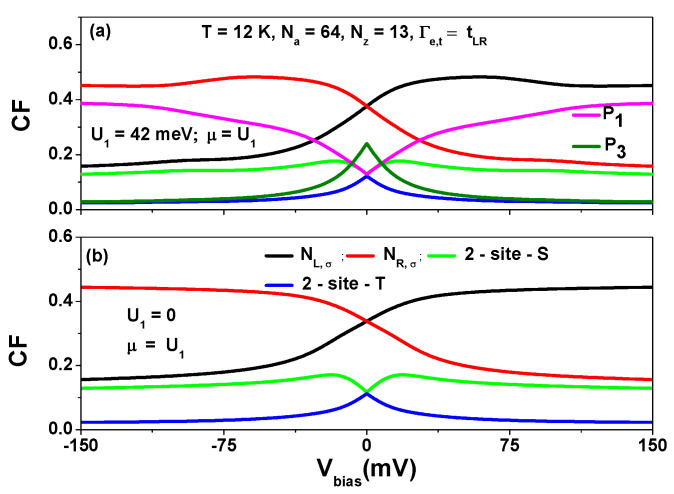
The correlation functions (CF) of AGNRs with $N_{a}=64$ and $N_{z}=13$ at $E_{L}=E_{R}=0$, $\mu =U_{1}$ meV, $\Gamma_{e,t}=t_{LR}$ and T= 12K, are plotted as a function of applied bias in (**a**) $U_{1}=42$ meV and (**b**) $U_{1}=0$. It should be noted that $\mu_{L}=\mu +eV_{bias}/2$ and $\mu_{R}=\mu -eV_{bias}/2$ were used in the calculation of the tunneling current.

In Figure 9b, the NDC behavior disappears when we artificially set the inter-site Coulomb interactions to zero ($U_{1}=0$) and only take into account the intra-site Coulomb interactions ($U_{0}=155$ meV). The plateau of the tunneling current is attributed to the intra-site Coulomb interaction of $U_{0}$, as $U_{1}$ is zero. It is worth noting that in Figure 9a,b, we do not consider the effect of bias-dependent orbital offset. Therefore, such NDC characteristics are not a result of off-resonant channels introduced by applied bias [38]. To clarify the NDC behavior shown in Figure 9a, we added two curves in Figure 9a, which were calculated considering the $P_{1}$ and $P_{3}$ channels of Equation (6), respectively. The $P_{3}$ channel plays an important role for $\left|V_{bias}\right|<50$ mV. However, it should be noted that the tunneling current is dominated by the $P_{1}$ channel when $|V_{bias}|>70$ mV. From the curves of $P_{1}$ and $P_{3}$, we understand that the NDC behavior of the tunneling current is due to the reduction in the probability of the $P_{3}$ channel. In Figure A4, we provide the single-particle occupation number and two-site two-particle correlation functions, such as the inter-site triplet states $\langle n_{R,\sigma }n_{L,\sigma }\rangle$ ($\langle n_{L,\sigma }n_{R,\sigma }\rangle$) and singlet states $\langle n_{R,-\sigma }n_{L,\sigma }\rangle$ ($\langle n_{L,-\sigma }n_{R,\sigma }\rangle$), which determine the probabilities of the resonant channels $P_{1}$ and $P_{3}$ in $T_{LR}^{2-site}\left(\varepsilon \right)$ ($T_{RL}^{2-site}\left(\varepsilon \right)$).

**Figure 10:**
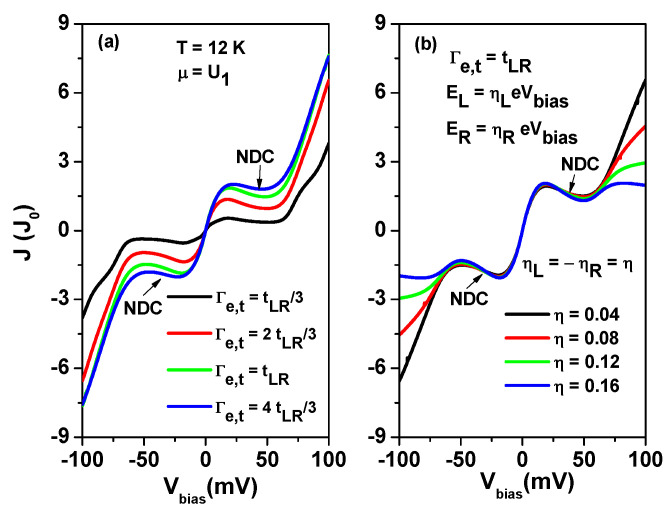
(**a**) Tunneling current as a function of $V_{bias}$ for various $\Gamma_{e,t}$ at T=12 Kand $E_{L}=E_{R}=0$, and (**b**) tunneling current as a function of $V_{bias}$ for various η values at T=12 K, $\Gamma_{e,t}=t_{LR}$ and $E_{L(R)}=\eta_{L(R)}eV_{bias}$. We have set $\mu =U_{1}=42$ meV. Other physical parameters are the same as those of Figure 9.

The electronic transport behavior can be significantly impacted by the properties of the contact between AGNRs and the electrodes [19,20,51,52,53,54]. Therefore, it is important to investigate the effect of the parameter $\Gamma_{e,t}$ on the NDC behavior. In Figure 10a, we present the tunneling current for different values of $\Gamma_{e,t}$ at T=12 K, where the variation of $\Gamma_{e,t}$ corresponds to finite AGNRs or AGNR heterostructures with different contact properties [53]. To analyze the effect of the applied bias on the orbital offset, we adopted the method of Ref. [55], where the bias-dependent energy level of TSs is given by $E_{L(R)}=\eta_{L(R)}\;eV_{bias}$ in Figure 10b. As shown in Figure 10a, the tunneling current increases as $\Gamma_{e,t}$ increases, whereas the NDC region is reduced. Figure 10b shows that the tunneling current is significantly suppressed in the region of $|V_{bias}|>50$ mV when the bias-dependent orbital offset is taken into account. We observed that a second NDC region appears at high applied bias for η=0.16.

**Figure 11:**
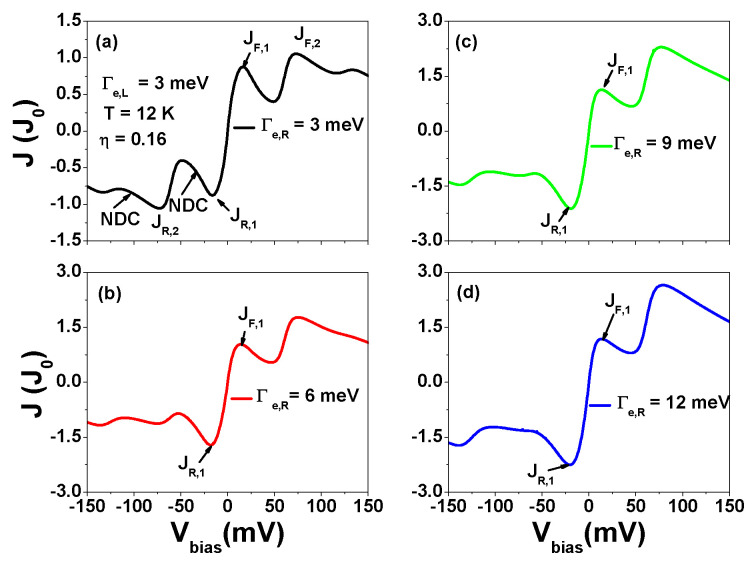
Tunneling current as a function of $V_{bias}$ for various $\Gamma_{e,R}$ values at $\Gamma_{e,L}=3$ meV, T=12 K and $\eta_{L}=-\eta_{R}=0.16$. $\mu =U_{1}=42$ meV. (**a**) $\Gamma_{e,R}=3$ meV, (**b**) $\Gamma_{e,R}=6$ meV, (**c**) $\Gamma_{e,R}=9$ meV and (**d**) $\Gamma_{e,R}=12$ meV. Other physical parameters are the same as those of Figure 9.

**Figure A5:**
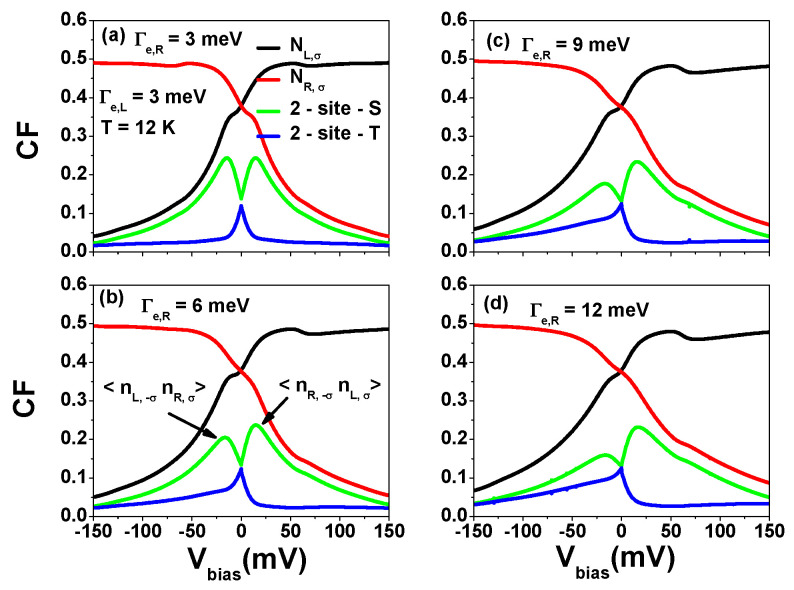
Correlation functions CF as a function of applied bias at $E_{L}=E_{R}=0$, $\mu =U_{1}=42$ meV, η=0.16, $\Gamma_{e,L}=3$ meV and T=12 K. (**a**) $\Gamma_{e,R}=3$ meV, (**b**) $\Gamma_{e,R}=6$ meV, (**c**) $\Gamma_{e,R}=9$ meV, and (**d**) $\Gamma_{e,R}=12$ meV. Other physical parameters are the same as those of Figure 11.

Up until now, our calculations assumed a symmetrical contact junction with $\Gamma_{e,L}=\Gamma_{e,R}$. However, in Figure 11, we present tunneling current results for $\Gamma_{e,L}\neq \Gamma_{e,R}$. In addition to NDC, we also observed current rectification behavior. The maximum current values in the low forward and reversed applied bias are denoted as $J_{F,1}$ and $J_{R,1}$, respectively. The ratio of $|J_{R,1}/J_{F,1}|$ increases with increasing $\Gamma_{e,R}$ when $\Gamma_{e,L}=3$ meV. It reaches 1.91 for $\Gamma_{e,R}=12$ meV. As discussed in Figure 9, $J_{F,1}$ and $J_{R,1}$ are primarily attributed to the $P_{3}$ channel of Equation (6). The probability weight of $P_{3}$ is primarily determined by the single-particle occupation number $N_{R,\sigma }=\langle n_{R,\sigma }\rangle$ ($N_{L,\sigma }=\langle n_{L,\sigma }\rangle$) and the two-site singlet state $\langle n_{R,-\sigma }n_{L,\sigma }\rangle$ ($\langle n_{L,-\sigma }n_{R,\sigma }\rangle$). When $|V_{bias}|<150$ mV, the intra-site two-particle occupation number $\langle n_{R,-\sigma }n_{R,\sigma }\rangle$ ($\langle n_{L,-\sigma }n_{L,\sigma }\rangle$) and two-site three-particle correlation functions are negligible. In the case of $\Gamma_{e,L}=\Gamma_{e,R}$, we have $N_{L,\sigma }\left(V_{bias}\right)=N_{R,\sigma }\left(-V_{bias}\right)$ (see Figure A5). In contrast, if $\Gamma_{e,L}<\Gamma_{e,R}$, then $N_{L,\sigma }\left(V_{bias}\right)\neq N_{R,\sigma }\left(-V_{bias}\right)$. Meanwhile, the two-site singlet state (2-site-S) in the reversed bias region is smaller than that in the forward bias region. Therefore, we observe that $|J_{R,1}|$ is larger than $J_{F,1}$. Note that if the inter-site Coulomb interaction $U_{1}$ is set to zero, the current rectification is significantly reduced as $\Gamma_{e,L}\neq \Gamma_{e,R}$. This suggests that the inter-site Coulomb interactions play a crucial role in determining the behavior of negative differential conductance (NDC) and current rectification. Note that the nonlinear current-voltage characteristics (NDC) and current rectification behavior discussed for a finite AGNR can also be observed in an AGNR heterostructure.

### 3.4. Tunneling Current through TSs of 9-7-9 AGNR Heterostructures under Pauli Spin Blockade Configuration

**Figure 12:**
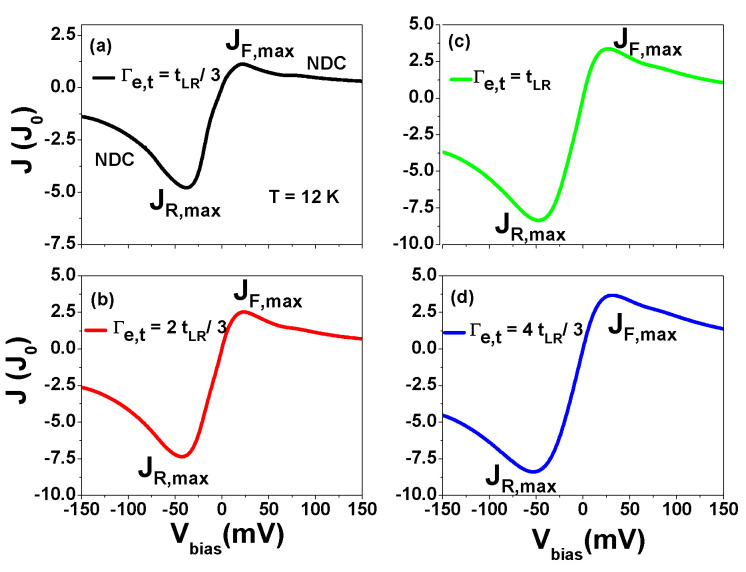
Tunneling current through the topological states (TSs) of 9-7-9 AGNR heterostructures in the Pauli spin blockade configuration as a function of $V_{bias}$ for various $\Gamma_{e,t}$ values at T=12 K. The energy levels of the left and right TSs are given by $E_{L}=-U_{1}+\eta_{L}eV_{bias}$ and $E_{R}=-U_{0}+\eta_{R}eV_{bias}$, respectively, where μ=0 meV and $\eta_{L}=-\eta_{R}=0.16$. (**a**) $\Gamma_{e,t}=t_{LR}/3$, (**b**) $\Gamma_{e,t}=2t_{LR}/3$, (**c**) $\Gamma_{e,t}=t_{LR}$ and (**d**) $\Gamma_{e,t}=4t_{LR}/3$. The parameters used are $U_{0}=125$ meV, $U_{1}=49$ meV, and $t_{LR}=7.54$ meV, which correspond to AGNR heterostructures formed by eight u.c. segments.

Because the topological states (TSs) of 9-7-9 AGNR heterostructures are located at the interfaces between 9-7 junctions, they can be far away from the contacted electrodes based on the design (refer to Appendix A or Figure 1b). Therefore, it is possible to arrange the two gate electrodes in a way that allows for the modulation of the energy levels of TSs and setting them in the Pauli spin blockade (PSB) configuration. We present the calculated tunneling current through the TSs of 9-7-9 AGNR heterostructures in the PSB configuration as a function of $V_{bias}$ for various $\Gamma_{e,L}=\Gamma_{e,R}=\Gamma_{e,t}$ values at T=12 K in Figure 12, where each AGNR segment has eight unit cells. Such TSs have $U_{0}=125$ meV, $U_{1}=49$ meV and $t_{LR}=7.54$ meV. As seen in Figure 12, a remarkable current rectification behavior is observed in the PSB configuration at the symmetrical tunneling rate $\Gamma_{e,L}=\Gamma_{e,R}$. The ratio of $|J_{R,max}/J_{F,max}|$ are 4.242, 2.908, 2.477 and 2.286 for $\Gamma_{e,t}=t_{LR}/3$, $\Gamma_{e,t}=2t_{LR}/3$, $\Gamma_{e,t}=t_{LR}$ and $\Gamma_{e,t}=4t_{LR}/3$, respectively.

To understand the rectification behavior shown in Figure 12, we first note that under PSB, the channel $P_{2}$ in Equation (6) behaves as a resonant channel in the forward applied bias, and the tunneling current is mainly contributed by $P_{2}$. We therefore focus on the transmission coefficient of $P_{2}$ in the PSB configuration, which can be expressed as:

$$\begin{array}{ccc} T_{PSB}\left(\varepsilon \right) & = & \frac{P_{PSB}}{|\left(\varepsilon -E_{L}-U_{1}+i\Gamma_{e,L}\right)\left(\varepsilon -E_{R}-U_{0}+i\Gamma_{e,R}\right)-t_{LR}^{2}{|}^{2}}, \end{array}$$

**Figure 13:**
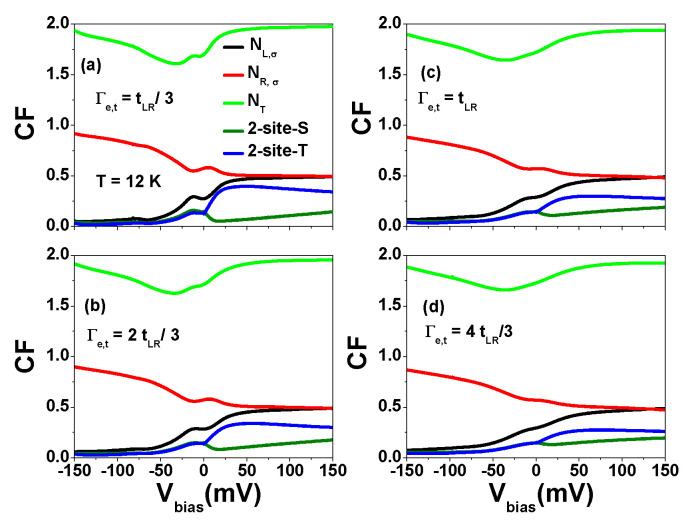
Correlation functions (CF) of 9-7-9 AGNR heterostructures in the PSB for various $\Gamma_{e,t}$ values at T=12 K. (**a**) $\Gamma_{e,t}=t_{LR}/3$, (**b**) $\Gamma_{e,t}=2t_{LR}/3$, (**c**) $\Gamma_{e,t}=t_{LR}$ and (**d**) $\Gamma_{e,t}=4t_{LR}/3$. Other physical parameters are the same as those of Figure 12.

Here, the probability of the resonant channel is given by $P_{PSB}=N_{R,\sigma }-\langle n_{R,\sigma }n_{L,\sigma }\rangle -\langle n_{R,-\sigma }n_{R,\sigma }\rangle +\langle n_{R,-\sigma }n_{R,\sigma }n_{L,\sigma }\rangle$ ($P_{PSB}=N_{R,\sigma }-\langle n_{L,\sigma }n_{R,\sigma }\rangle -\langle n_{L,-\sigma }n_{R,\sigma }\rangle +\langle n_{L,-\sigma }n_{L,\sigma }n_{R,\sigma }\rangle$) in the forward (reversed) applied bias. In the small applied bias region $\left|V_{bias}\right|\leq 50$ mV, the resonant channels are almost unaffected by bias-dependent orbital offset, and $P_{PSB}$ is the main factor determining the magnitude of the tunneling current. For $\left|V_{bias}\right|\leq 150$ mV, only single occupation number and inter-site singlet and triplet states are important. We show these correlation functions in Figure 13 to clarify the bias-dependent $P_{PSB}$.

As seen in Figure 13a, charge transport in the PSB region is a two-electron process because of the total electron number $1.5\leq N_{T}\leq 2$. In the forward applied bias, $N_{R,\sigma }$ and $N_{L,\sigma }$ are in discharging and charging processes, respectively, and their values reach 0.5 at $V_{bias}=150$ mV. Due to the large probability of inter-site triplet state (see the curve of 2-site-T), the $P_{PSB}$ is degraded. In contrast, $N_{R,\sigma }$ and $N_{L,\sigma }$ in the reversed applied bias are in the charging and discharging processes, respectively. The highly enhanced $N_{R,\sigma }$ and suppressed inter-site correlation functions (see 2-site-T and 2-site-S) result in a large $P_{PSB}$. This explains the significant current rectification observed in Figure 12a. With increasing $\Gamma_{e,t}$, the magnitude of 2-site-T is degraded for $V_{bias}>0$, leading to an enhancement of $J_{F,max}$ and a reduction in $|J_{R,max}/J_{F,max}|$ in Figure 12. In addition, we observe an interesting behavior of phase transformation. The merge together 2-site-T and 2-site-S curves in the reversed applied bias region is splitting into two curves in the forward applied bias.

**Figure 14:**
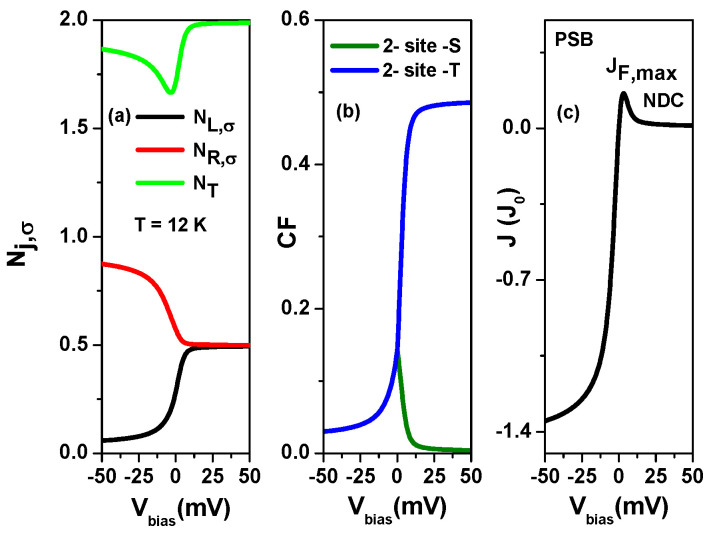
(**a**) Single-particle occupation numbers $N_{j,\sigma }$, (**b**) inter-site two-particle correlation functions (CF), and (**c**) tunneling current of 9-7-9 AGNR heterostructures as functions of μ for a small $t_{LR}=0.88$ meV at T=12 K, $\Gamma_{e,L}=t_{LR}/3$, and $\Gamma_{e,R}=3t_{LR}$. We realize the small $t_{LR}=0.88$ meV using 12 u.c. segments to form 9-7-9 AGNR heterostructures. Their intra-site and inter-site Coulomb interactions are $U_{0}=111$ meV and $U_{1}=36.97$ meV. The energy levels of TSs are $E_{L}=-U_{1}$ and $E_{R}=-U_{0}$.

Finally, we examine SCTSs in the PSB configuration for weak coupling strength of $t_{LR}$ values. In Figure 14, we present (a) single-particle occupation numbers $N_{j,\sigma }$, (b) inter-site two-particle correlation functions, and (c) tunneling current of 9-7-9 AGNR heterostructures as functions of μ at T=12 K, $t_{LR}=0.88$ meV, $\Gamma_{e,L}=t_{LR}/3$, and $\Gamma_{e,R}=3t_{LR}$. To achieve the small coupling parameter of $t_{LR}=0.88$ meV, we use 12 u.c. segments to form 9-7-9 AGNR heterostructures. We can disregard the bias-dependent orbital offset between TSs, as the wave functions of TSs are distant from the electrodes and the channel length between the electrodes is significantly extended [55]. In Figure 14a, the total electron number $N_{T}$ is constrained within the range of $1.5<N_{T}\leq 2$. As observed in Figure 14b, the 2-site-T curve reaches its maximum value of 0.486, while the 2-site-S curve approaches nearly zero in the forward applied bias. When compared to the results in Figure 13, it is apparent that the forward applied bias and weak $t_{LR}$ lead to high occupancy of inter-site triplet states in SCTSs. Consequently, the SCTSs occupied by triplet states generate an extremely small current ($J_{F}$) as shown in Figure 14c. The significant current rectification demonstrated in the PSB configuration, as depicted in Figure 14, holds great value for spin-current conversion devices [11].

## 4. Conclusions

In summary, this study provides an in-depth investigation of the charge transport properties of two distinct graphene nanoribbon (GNR) structures: the end zigzag edges of AGNRs and the topological states of 9-7-9 AGNR heterostructures. Our findings demonstrate that 9-7-9 AGNR heterostructures with deep topological states (TSs) exhibit superior characteristics, such as wave functions of TSs far away from the electrodes, making them more suitable for low-power quantum devices. The two-site model with effective tunneling rates provides an excellent description of the electrical conductance spectra of the serially coupled topological states (SCTSs). Additionally, we analyzed the Coulomb blockade effect on the thermoelectric coefficients and tunneling current of the zigzag edge states using the two-site Hubbard model. Our results show that electron Coulomb interactions have a more significant impact on electrical conductance than on the Seebeck coefficient. The Lorenz number of the zigzag edge states does not satisfy the Wiedemann–Franz law due to their localized characteristic.

We observed negative differential conductance (NDC) in the nonlinear response regime of the tunneling current, attributed to inter-zigzag edge Coulomb interactions. Additionally, we observed current rectification behavior in the end zigzag edges of AGNRs when asymmetrical contacted electrode junctions were present. The tunneling current through SCTSs formed by 9-7-9 AGNR heterostructures in the Pauli spin blockade configuration exhibits remarkable current rectification behavior, even in AGNR heterostructures with symmetrical contacted electrodes. For a weak coupling parameter $t_{LR}$, the forward tunneling current is almost blocked due to the high occupation of SCTSs by two-electron triplet states. This property is highly useful in spin-current conversion devices. Overall, our study provides valuable insights into the charge transport properties of TSs in finite AGNRs and heterostructures, emphasizing the importance of considering electron-electron interactions in understanding their behavior.

## Figures and Tables

**Figure A1:**
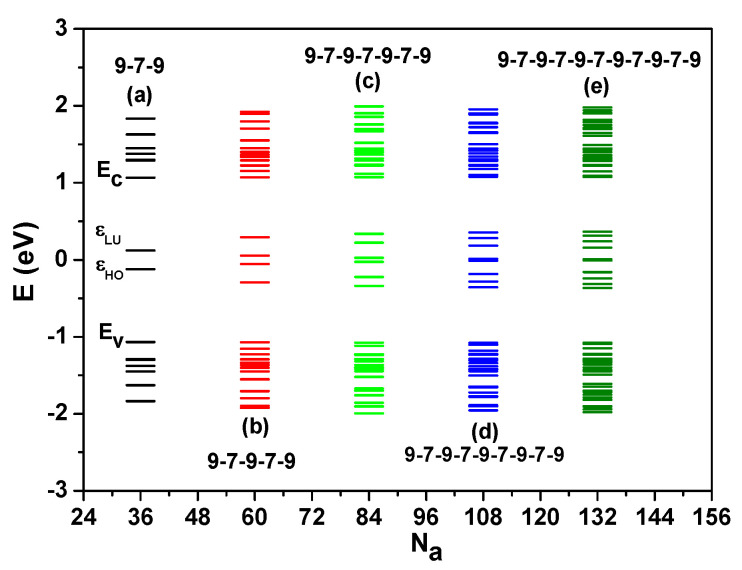
Eigenvalues of AGNR heterostructures with varying numbers of 7-AGNR segments. 9-7-9 AGNR heterostructures are formed by 3 u.c segments. (**a**–**e**) correspond to one to five 7-AGNR segments, respectively, with an energy range of |E|≤2 eV.

**Figure A2:**
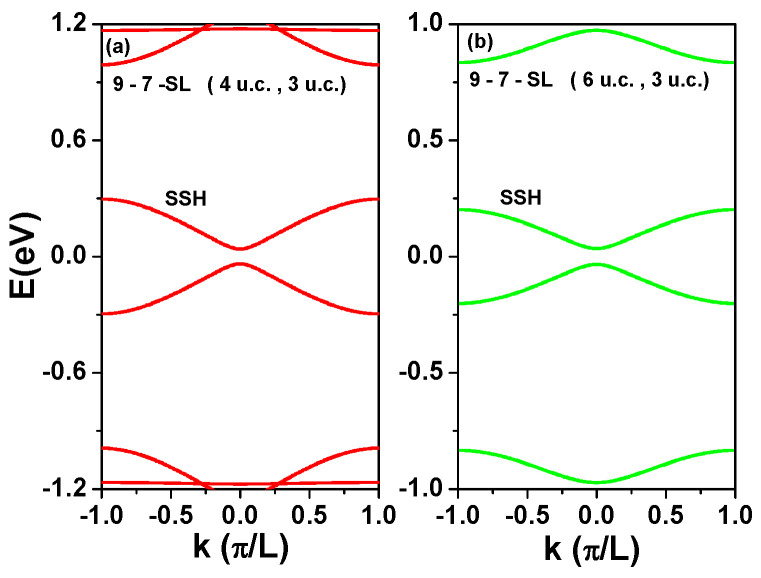
Electronic structures of 9-7 AGNR superlattices (SLs). (**a**,**b**) correspond to 9-AGNR segments with 4 u.c. and 6 u.c., respectively, with an energy range of |E|≤1.2 eV. Note that the 7-AGNR segment with 3 u.c. is fixed. *L* is the length of the super unit cell. We have L=7 u.c. and L=9 u. c. in (**a**,**b**), respectively.

**Figure A3:**
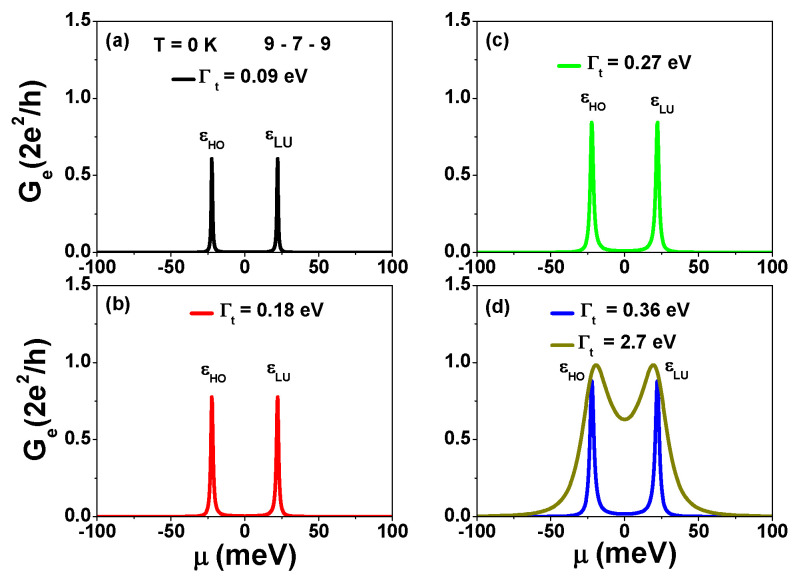
Electrical conductance of 9-7-9 AGNR heterostructures formed by 6 u.c. segments as functions of chemical potential (μ) for various $\Gamma_{t}$ values at T=0 K. (**a**) $\Gamma_{t}=0.09$ eV, (**b**) $\Gamma_{t}=0.18$ eV, (**c**) $\Gamma_{t}=0.27$ eV and (**d**) $\Gamma_{t}=0.36$ eV. We added an extra curve of $\Gamma_{t}=2.7$ eV in (**d**).

## Data Availability

The data presented in this study are available upon reasonable request.

## Appendix A. 9-7-9 AGNR Heterostructures

The results presented in Figure 2b indicate that the wave function of the left zigzag edge state has only a small overlap with that of the right zigzag edge state for the case of $N_{z}=9$. Consequently, it is difficult for electrons from the electrodes to be transported through these localized states. Recently, interesting topological states (TSs) have been identified in the electronic structures of 9-7 AGNR heterostructures. In these structures, the wave function of TS accumulates at the interface between the 9-7 junction [17,18,19,20]. However, the charge transport through these serially coupled TSs (SCTSs) has not been clarified [38,51,52]. In the Appendix A, we not only further clarify their electronic structures but also investigate how the contacted electrodes influence the charge transport through the SCTSs.

As depicted in Figure 1b, the 9-7-9 AGNRs are formed by three u.c. segments. The calculated eigenvalues of the AGNR heterostructures for various periods are shown in Figure A1. As seen in the case of (a), two peaks labeled by $\varepsilon_{HO}$ and $\varepsilon_{LU}$ with a splitting energy of $|2t_{LR}|=0.24312$ eV between $E_{c}$ and $E_{v}$ are observed. Here, $E_{c}$ and $E_{v}$ denote the conduction subband minimum and valence subband maximum, respectively. The charge density distribution of $\varepsilon_{LU}=0.12156$ eV is presented in Figure 1b, which shows that the charge densities are very dilute for lattices close to the end zigzag edges. Such behavior indicates that $\varepsilon_{HO}$ and $\varepsilon_{LU}$ have weak coupling strengths with the electrodes. As each 7-AGNR segment provides two energy levels, there are 4, 6, 8, and 10 energy levels within the band gap for (b), (c), (d), and (e), respectively, as shown in the results. When the number of 7-AGNR segments approaches infinity, the minibands are formed. To reveal such interesting electronic structures formed by TSs, we show the calculated electronic structures of 9-7 AGNR superlattices (SLs) in Figure A2, where (a) and (b) consider different 9-AGNR segment lengths at fixed three u.c. 7-AGNR segments. Two minibands are formed near the charge neutrality point (CNP), the tiny gaps in (a) and (b) are 76 meV and 68 meV, respectively. We find that the electronic dispersions of minibands can be well described by the Su–Schrieffer–Heeger (SSH) model with a closed expression of $E_{SSH}\left(k\right)=\pm \;\sqrt{t_{1}^{2}+t_{2}^{2}-2t_{1}t_{2}cos\left(k\;\pi /L\right)}$ [16,19,54]. Using $E_{SSH}\left(k\right)$, we can determine $t_{1}=0.167$ eV and $t_{2}=0.129$ eV in (a) and $t_{1}=0.118$ eV and $t_{2}=0.084$ eV in (b). As seen in Figure A2, the miniband widths become narrow as the length of the 9-AGNR segment increases. According to the SSH model, the metal, semiconductor, and insulator phases are determined by the relationships between $t_{1}$ and $t_{2}$. Once $t_{1}=t_{2}$, the band gap is vanishingly small.

The charge densities of the topological states (TSs) in 9-7-9 AGNR heterostructures are distinct from those of the end zigzag edge states found in finite AGNRs. The wave functions of the TSs are located far away from the line-contacted electrodes, which allows for the observation of charge transport through the TSs of 9-7-9 heterostructures even at large coupling strengths between the zigzag edge carbon atoms and the electrodes (i.e., large $\Gamma_{t}$ values). We calculated the electrical conductance of 6-unit cell (u.c.) 9-7-9 AGNRs for various $\Gamma_{t}$ values, and the results are presented in Figure A3. The splitting energy between the highest occupied ($\varepsilon_{HO}$) and lowest unoccupied ($\varepsilon_{LU}$) states is $|2t_{LR}|=44.4$ meV, and their magnitudes and widths increase with increasing $\Gamma_{t}$. Notably, even when $\Gamma_{t}=2.7$ eV, the two peaks of $\varepsilon_{HO}$ and $\varepsilon_{LU}$ are still clearly resolved. The excellent agreement between the results of Figure A3 and Equation (7) (not shown here) is due to the TSs’ localized wave functions. Therefore, a 2-site Hubbard model is also suitable for describing charge transport through the TSs of 9-7-9 AGNR heterostructures.

## Appendix B. Correlation Functions of the End Zigzag Edges of AGNRs

In Figure 9, we illustrated the tunneling current of the end zigzag edge states as a function of $V_{bias}$ for various temperatures. The behavior of the tunneling current is determined by the resonant channels and their probabilities. Since $E_{L}$ and $E_{R}$ do not have a bias-dependent offset, the NDC behavior arises from the bias-dependent probabilities. Therefore, it is essential to understand the behavior of $P_{n}$ in Equation (6). Figure A4 displays the single-particle occupation number $N_{L(R),\sigma }=\langle n_{L(R),\sigma }\rangle$ and inter-site two-particle correlation functions (CF) as functions of the applied bias ($V_{bias}$), with and without inter-site Coulomb interactions. Due to the symmetrical structure of the junction system, we have $N_{L,\sigma }\left(V_{bias}\right)=N_{R,\sigma }\left(-V_{bias}\right)$. The 2-site singlet (2-site-S) state $\langle n_{R,-\sigma }n_{L,\sigma }\rangle$ and 2-site triplet (2-site-T) state $\langle n_{R,\sigma }n_{L,\sigma }\rangle$ (or $\langle n_{L,-\sigma }n_{R,\sigma }\rangle$ and $\langle n_{L,\sigma }n_{R,\sigma }\rangle$) also exhibit this symmetric character with respect to $V_{bias}$. We analyzed their behavior in the case of a forward bias situation ($V_{bias}>0$). $N_{L(R),\sigma }$ has a finite value at zero bias ($V_{bias}=0$ mV) since $E_{L}$ and $E_{R}$ are below μ. As $V_{bias}$ increases, $N_{L,\sigma }$ increases, but $N_{R,\sigma }$ decreases. Eventually, $N_{R,\sigma }$ will saturate. The charge filling of $E_{R}$ by the left electrode is attributed to the resonant channel between $E_{L}$ and $E_{R}$. Since $N_{R,\sigma }$ is indirectly charged by the left electrode, in the forward applied bias range, $N_{R,\sigma }$ is smaller than $N_{L,\sigma }$. We found that the probability of 2-site-S is larger than that of 2-site-T at finite bias. The bias-dependent probabilities of $P_{1}$ and $P_{3}$ channels, which provide resonant energy levels, are plotted in Figure A4a. The decline behavior of the probability of $P_{3}$ explains the behavior of the tunneling current with NDC shown in Figure 9.

To understand the current rectification behavior observed in Figure 11, we present $N_{L(R),\sigma }$ and the inter-site two-particle correlation functions (2-site-S and 2-site-T) for various $\Gamma_{e,R}$ values at $\Gamma_{e,L}=3$ meV in Figure A5. As shown in Figure A5, the condition of $N_{L,\sigma }\left(V_{bias}\right)=N_{R,\sigma }\left(-V_{bias}\right)$ is lifted when the junctions have asymmetrical contacted properties. $P_{3}$ channels dominate the behavior of the tunneling current at low bias. Therefore, the forward maximum current ($J_{F,1}$) and the reversed maximum current ($J_{R,1}$) can be determined by $P_{3}=N_{R,-\sigma }-\langle n_{R,-\sigma }n_{L,\sigma }\rangle$ and $P_{3}=N_{L,-\sigma }-\langle n_{L,-\sigma }n_{R,\sigma }\rangle$, respectively. In the low bias region, we have $N_{L,\sigma }\approx N_{R,\sigma }$ and $\langle n_{R,-\sigma }n_{L,\sigma }\rangle >\langle n_{L,-\sigma }n_{R,\sigma }\rangle$, which explains why $J_{R,1}$ is larger than $J_{F,1}$ and the current rectification behavior is observed in Figure 11.
